# Fibroid Uterus

**Published:** 1914-03

**Authors:** W. H. C. Newnham

**Affiliations:** Physician Accoucheur, Bristol General Hospital


### FIBROID UTERUS.

BY

W. H. C. NEWNHAM, M.A., M.B. Cantab.,
*Physician Accoucheur, Bristol General Hospital.*

IT is not possible in surgery to point with greater triumph to any operation than intra-peritoneal hysterectomy, when one thinks that only so lately as 1896 the operation was performed by very few surgeons on account of its very high mortality, but from 1896 to 1906 it came down to 2 per cent.

After many journeys to London and watching the operation most carefully, I undertook the first in Bristol at the General Hospital in 1898. It was quite successful, and I brought the case forward at a meeting of the Bristol Medico-Chirurgical Society; but the operation was criticised by those who preferred to keep to the old operation of transfixing the stump outside the wound by means of the serre-nœud, and allowing the last piece of the uterus to slough off by a daily tightening of the surrounding wire. Of course, in many of these cases suppuration followed, and the patient's life was in jeopardy, and I ventured to predict that this, the extra-peritoneal procedure, must become obsolete, and that the then new intra-peritoneal operation for all fibroids was the treatment that had come to stay.

In 1906 there were 348 hysterectomies recorded in London, with eleven deaths, while in 1906–7 Bland Sutton did 101 cases without a single death.

It will thus be seen that removal of the uterus for fibroids by the abdominal route intra-peritoneally is a fairly safe proceeding; nevertheless hysterectomy, like all other major surgical operations, is attended with considerable risk, and should only be undertaken by those who have had opportunities for gaining adequate experience and practice.

Baer's method of supra-vaginal hysterectomy is far the safer and easier operation, but some surgeons prefer the total on account of the idea that possibly cancer may occur in the vaginal stump. If this ever should occur, it would probably be found that cancer was present in the neck of the uterus at the time of the operation, or that the tumour removed for fibroid was really sarcomatous or malignant. The strongest advocates of total hysterectomy for fibroids are probably those who have had but a limited experience of the total operation. I have not yet seen in my large number of cases any sign of malignancy in any operation that I have undertaken for fibroids.

It is generally admitted now that it is better if possible to preserve both ovaries or at least one in doing this operation, and not remove them as Baer originally did.

The advantages of leaving them are many, but the most important is that it prevents the severity of an artificial menopause.

In regard to marital relations, I have had many proofs that the preservation of one or both ovaries promotes domestic bliss. Also many spinsters have married after this operation, and yet have been able to lead perfectly happy lives from their own point of view and also that of their husbands.

The belief that fibroids disappear at the menopause is now, I think, quite abandoned ; my own experience would tell me that if anything they have a tendency to grow rather faster after it than before, and many patients that I saw ten or twelve years ago, and on whom I did not urge an operation, have come to me with such large tumours that the abdominal cavity is quite filled with the growth.

A fibroid lodged in a pregnant uterus is very liable to undergo red degeneration and become very painful, whereas ordinary fibroids are not usually associated with any pain.

I have been compelled to do two myomectomies for red degeneration of a fibroid in a pregnant uterus on account of the excessive pain. On each occasion I was able to shell out the tumour without affecting the uterus, and pregnancy was successfully continued up to the ninth month in each case.

Like all big operations connected with the reproductive organs, hysterectomy for fibroids has had to pass through a period of prejudice. At the present time no major operation can show such a low mortality or produce such permanent good effects.

A wife spending half her life as a chronic invalid, in bed half the month with hemorrhage, only able to get about the other half in a wheel-chair, is able now to become a companion to her husband, while a spinster may be enabled to earn her own living instead of being a helpless invalid. Such is the success of this operation, that I am inclined to lay down the law and say, " No matter how small the fibroid may be, when you have once found it, it is better to advise the patient to have it removed." There is then no risk of complications, or of being blamed afterwards for not giving this advice if any accident happens. It must be remembered that though it is possible for a woman to have a fibroid and be unconscious of the fact, still in the majority of cases they are the cause of much suffering and may cause death in insidious ways.

Hemorrhage, of course, is one of the chief symptoms, and we must remember that if a woman bleeds excessively at the periods and also between them, it is quite likely that the tumour has become septic, and if this is allowed to continue for any length of time it will surely destroy the life of its owner.

Pyosalpinx may often be found as a complication of uterine fibroids when they have been left to become septic, and I have operated upon four cases with this complication, luckily with good results in all of them.

When a fibroid occupies the posterior wall of the uterus it often causes retroflexion of the body and fills up the pelvis, and often I have found some difficulty in pulling up the tumour owing to this complication. When a woman complains of retention of urine for a few days before the menstrual period at the ages of 35 to 45, then look out for a fibroid in the uterus.

I find that on looking through my book I have operated on a series of 119 fibroids with only one death.

